# Slits in the wall! Pulvinar slits confer unique cell wall mechanics for leaf movement

**DOI:** 10.1093/plphys/kiad166

**Published:** 2023-03-18

**Authors:** Dyoni M Oliveira

**Affiliations:** Assistant Features Editor, Plant Physiology, American Society of Plant Biologists, USA; Department of Plant Biotechnology and Bioinformatics, Ghent University, Ghent 9052, Belgium; VIB Center for Plant Systems Biology, Ghent 9052, Belgium

**Figure 1.**
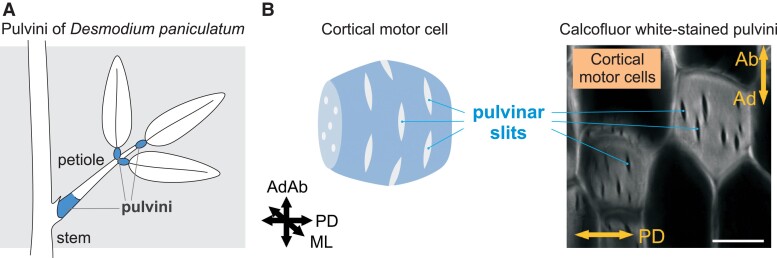
Pulvinus and pulvinar slits of *D. paniculatum*. **A)** Scheme of pulvini regions. **B)** Proposed model of pulvinar slits in the wall of a cortical motor cell (left). Section of calcofluor white-stained *D. paniculatum* pulvini visualized by confocal laser scanning microscopy (right). Bar, 20 *µ*m. AdAb, adaxial–abaxial axis; ML, medial–lateral axis; PD, proximo-distal axis. **B)** was modified from Figure 1C and G of Takahara et al. (2023).

Vascular plants exhibit reversible leaf movements that can take place over timescales from seconds (e.g. folding in mimulus) to hours (circadian movements). Many of these reversible movements are mediated by the pulvinus, a specialized organ at the base of the leaf, leaflet, petiole, or petiolule (Fig. 1A). The cortical motor cells form the swelling part of the pulvinus and execute the growth-independent motion and the bending of leafy appendages (Nakata and Takahara 2022). During leaf movement, pulvinus motion is associated with the active transport of ions from parenchyma cells and sugars from vascular elements, resulting in fluid movement and turgor-induced cell deformation (Kumon and Suda 1984; Sleboda et al. 2023).

The cell wall is a dynamic and responsive structure that sustains cell division and expansion, provides structural integrity to each cell, and serves in sensing environmental changes (Oliveira et al. 2020b; Coen and Cosgrove 2023). Cellulose, hemicelluloses, and pectins are the main components of primary cell walls. How do the wall composition and properties of cortical motor cells contribute to the leaf motion has remained a mystery, however. Recent advancements have provided important evidence of cellulose–cellulose bonding and noncovalent cellulose–pectin interactions in the primary walls of Arabidopsis (*Arabidopsis thaliana*) (Wang et al. 2015; Cosgrove 2018); however, the intra- and intermolecular cross-links between cellulose microfibrils with xyloglucan hemicellulose and pectins are still unclear.

In this issue of *Plant Physiology*, Takahara et al. (2023) set out to investigate the cell wall structure of cortical motor cells in the pulvinus of panicled-leaf ticktrefoil (*Desmodium paniculatum*). As it is not a model plant like Arabidopsis or maize (*Zea mays*), little is known about its cell wall makeup. The starting point for the authors’ work was the identification of the cell wall structure of cortical motor cells by staining pulvini with calcofluor white, which stains cellulose. Notably, the cortical motor cells presented several slits with no fluorescence in a circumferential orientation in the adaxial/abaxial-mediolateral plane, indicating a lower deposition of cellulose in slits. To determine whether the slits are specific to cortical motor cells, they also stained cortical cells from the petiole and from the boundary region between pulvinus and petiole. Both cases showed no slits. The authors, thus, named the circumferential slits in pulvinus cortical motor cells as “pulvinar slits” (Fig. 1B).

The authors wondered to what extent the pulvinar slits are conserved across different legumes with similarly dynamic leaves. To investigate this, they stained vertical sections of pulvini from 11 legume species with calcofluor white and confirmed the presence of slits in both adaxial and abaxial pulvinar cortical motor cells. These results demonstrated that pulvinar slits are innate wall components of cortical motor cells. Consistent with the observation that pulvinar slits are regions deficient in cellulose compared with the surrounding regions, Takahara et al. turned to cellulose-specific staining with pontamine and immunolabeling to confirm that slits have lower cellulose deposition. They concluded that pulvinar slits are regions of low cellulose deposition in the cell walls of both adaxial and abaxial cortical motor cells and are widely conserved in legumes. Interestingly, Sleboda et al. (2023) independently also reported the presence of primary pit fields in pulvini of *Mimosa pudica*, the structures of which have similar shape and size to pulvinar slits. Therefore, these observations suggest that pulvinar slits in *D. paniculatum* and primary pit fields in *M. pudica* might have analogous functions in the mechanics of the pulvinus.

In primary cell walls, the interaction of pectin and xyloglucan contributes to cell wall extensibility by interacting with cellulose microfibrils (Wang et al. 2015; Cosgrove 2018). Rhamnogalacturonan I and II and homogalacturonan are the main pectins, although the number and type of unique pectin structures in the wall remain unknown. Given that homogalacturonan is methyl-esterified or de-methyl-esterified according to the cell wall assembly (Wolf et al. 2009), the authors performed an immunohistological analysis in pulvini with a set of antibodies against homogalacturonan structures. The results indicated that pulvinar slits are rich in de-methyl-esterified homogalacturonan with no calcium associated with such structures, suggesting that de-esterified pectins might not require calcium-mediated cross-linkings for the mechanical regulation of cortical motor cells. Notably, the walls of stomatal guard cells are rich in de-esterified pectins, and stomatal opening and closure depends on the de-methyl-esterification of pectin in guard cell walls (Amsbury et al. 2016).

Next, they combined 2 orthogonal methods—Fourier-transform infrared (FTIR) spectroscopy and chemical analysis of monosaccharides released from cell walls—to determine the monosaccharide profiling of pulvinus cell walls and pectin-derived sugars. FTIR spectroscopy has been successfully used to further probe cell wall chemical fingerprints and distinguish wall chemotypes (Mota et al. 2019; Oliveira et al. 2020a). In this way, the authors evidenced that pulvinar organs have a distinct wall composition compared with petioles and developing stems in *D. paniculatum.* Additionally, monosaccharide profiling confirmed the high levels of galacturonic acid, likely from homogalacturonan, in pulvini.

Because pulvinar cell walls have a distinct chemotype and an abundant deposition of pectin relative to other axial organs, the authors expected to find differences in the mechanical properties of pulvini. They analyzed the proximo-distal extensibility under compression, in which the sample perpendicularly expands to the direction of compression (Cosgrove 2018). Pulvini displayed greater cell extensibility at the organ level compared with other axial organs. To determine whether the pulvinar slits affect the cell shape changes of cortical motor cells under turgor pressure, Takahara et al. used computational simulations via the finite-element method to specifically simulate how the elastic deformation is mediated by slits in the cell wall mechanics. The simulations indicated that size, density, and parallel alignment of pulvinar slits promote cell expansion under turgor pressure. Finally, a further analysis of large secondary pulvini from the legume species kudzu (*Pueraria montana* var. *lobata*) upon different extracellular osmotic conditions confirmed that pulvinar slits reversibly open and close following the turgor of cortical motor cells.

In summary, this study is a step forward in our current understanding of wall structure and mechanics, revealing that cell walls of cortical motor cells have pulvinar slits that have low levels of cellulose and are rich in de-methyl-esterified homogalacturonan. Furthermore, this discovery elicits new questions: What role does homogalacturonan play in pulvinar slits during the reversible leaf movement? Do the slits also occur in the walls of plants outside Fabaceae lineages? In fact, the independent evolution of components in plant cell walls is known. Equally important for future studies will be the investigation of the tissue-specific expression of genes associated with slit formation in pulvini.
